# Supplementary material for: Stochastic modeling of long-legged ant A. gracilipes locomotion in laboratory experiments
Source: PLoS Comput Biol. 2026 Aug 6;22(8):e1014069. doi: 10.1371/journal.pcbi.1014069 (PMC13446673; doi:10.1371/journal.pcbi.1014069)
Supplement: Supplementary file 1 [file pcbi.1014069.s004.pdf]

Appendix A: Fokker-Planck formulation

The Langevin formulation is useful for simulating trajectories of the stochastic process, but it is more convenient in the calculation of certain properties to work with the probability for the walker to be in any particular state, $P(\vec{x}, \theta, t)$.

We first write the Fokker-Planck (forward Kolmogorov equation) for a slightly simplified version of the stochastic process above, without finite tumbling times and in an infinite domain [1, 2]:

$$\frac{\partial}{\partial t}P = \underbrace{-(\vec{v}\cdot\vec{\nabla})P}_{\text{Runs}} + \underbrace{D_r\frac{\partial^2 P}{\partial\theta^2}}_{\text{Diffusion}} + \underbrace{\frac{1}{\tau}\left[\int d\theta' K(\theta-\theta')P(\vec{x},\theta',t) - P\right]}_{\text{Tumbles}}. \tag{1}$$

While the tumbling times are an important part of the real trajectories, they don't affect the spatial properties of the motion given our assumption that the ant doesn't move during these tumbling times. The infinite domain assumption is justified by only considering the trajectories away from the arena edges (see Discussion for further details). The first and second terms on the right side of Equation 1 implement the straight runs and the rotational diffusion behavior of the process, respectively. The final term controls the tumbling behavior, where the first part represents the increase in $P(\vec{x}, \theta, t)$ from the particle tumbling from some other angle θ' and ending up with θ. The latter term is the opposite, representing the decrease in P from particles tumbling to a different angle.

As in the main text, we assume a zero-centered von Mises angle kernel,

$$K(\Delta\theta) = \frac{1}{2\pi I_0(\sigma)}e^{\sigma\cos(\Delta\theta)}, \tag{2}$$

and run times (time between tumbles) that are Poisson distributed (already included in the Fokker-Planck above),

$$P_{\Delta t}(\Delta t) = \frac{1}{\tau}e^{-\Delta t/\tau}. \tag{3}$$

Persistence time of infinite run-and-tumble

Using the Fokker-Planck equation defined in the previous section, we derive here the persistence time of our run-and-tumble particle. This is done by computing the autocorrelation of the orientation, $\langle\cos\theta(t+\Delta t)\cos\theta(t)\rangle$. Assuming the process is stationary, we can simply consider how the cosine of the angle evolves through time, $\langle\cos\theta(\Delta t)\rangle$ This typically decays as an exponential, which allows us to extract the characteristic persistence time τ_p:

$$\langle\cos\theta(\Delta t)\rangle \sim e^{-\Delta t/\tau_p}. \tag{4}$$

In order to find the mean value of $\cos\theta$ as a function of time, we multiply Equation 1 by $\cos\theta(\Delta t)$ (abbreviated as $\cos\theta$) and integrate over the spatial and orientational degrees of freedom of $P(\vec{x}, \theta, t)$:

$$\int d\vec{x}\int d\theta\cos\theta\left[\frac{\partial}{\partial t}P = -(\vec{v}\cdot\vec{\nabla})P + D_r\frac{\partial^2 P}{\partial\theta^2} + \frac{1}{\tau}\left[\int d\theta' K(\theta-\theta')P(\vec{x},\theta',t) - P\right]\right]$$

$$\frac{\partial}{\partial t}\int d\theta P\cos\theta = -\int d\vec{x}\int d\theta(\vec{v}\cdot\vec{\nabla})P\cos\theta + D_r\int d\vec{x}\int d\theta\frac{\partial^2 P}{\partial\theta^2}\cos\theta$$
$$+\frac{1}{\tau}\left[\int d\vec{x}\int d\theta\int d\theta' K(\theta-\theta')P(\vec{x},\theta',t)\cos\theta - \int d\vec{x}\int d\theta P\cos\theta\right].$$

Since $\langle\cos\theta\rangle = \int d\vec{x}\int d\theta P\cos\theta$, the left hand side and the last term on the right hand side reduce easily to the (time derivative of the) desired mean:

$$\frac{\partial}{\partial t}\langle\cos\theta\rangle = -\int d\theta(\vec{v}\cdot\vec{\nabla})P\cos\theta + D_r\int d\vec{x}\int d\theta\frac{\partial^2 P}{\partial\theta^2}\cos\theta$$
$$+\frac{1}{\tau}\left[\int d\vec{x}\int d\theta\int d\theta' K(\theta-\theta')P(\vec{x},\theta',t)\cos\theta - \langle\cos\theta\rangle\right].$$

The diffusion term can be integrated by parts twice:

$$D_r\int d\vec{x}\int d\theta\frac{\partial^2 P}{\partial\theta^2}\cos\theta = D_r\int d\vec{x}\left[\cos\theta\frac{\partial P}{\partial\theta}\Big|_{-\pi}^{\pi} + \int d\theta\frac{\partial P}{\partial\theta}\sin\theta\right]$$
$$= D_r\int d\vec{x}\left[\cos\theta\frac{\partial P}{\partial\theta}\Big|_{-\pi}^{\pi} + \sin\theta P\Big|_{-\pi}^{\pi} - \int d\theta P\cos\theta\right].$$

Both of the boundary terms vanish, and the final term is simply the desired mean:

$$\frac{\partial}{\partial t}\langle\cos\theta\rangle = -\int d\theta(\vec{v}\cdot\vec{\nabla})P\cos\theta - D_r\langle\cos\theta\rangle$$
$$+\frac{1}{\tau}\int d\vec{x}\int d\theta\int d\theta' K(\theta-\theta')P(\vec{x},\theta',t)\cos\theta - \frac{1}{\tau}\langle\cos\theta\rangle.$$

The other tumble term can be evaluated by plugging in the form of K from Eq 2. Interchanging the integrals on θ and θ', we get:

$$\frac{1}{\tau}\int d\vec{x}\int d\theta\int d\theta' K(\theta-\theta')P(\vec{x},\theta',t)\cos\theta$$
$$= \frac{1}{\tau}\int d\vec{x}\int d\theta'\left[\int d\theta K(\theta-\theta')\cos\theta\right]P(\vec{x},\theta',t)$$
$$= \frac{1}{\tau}\int d\vec{x}\int d\theta'\left[\frac{1}{2\pi I_0(\sigma)}\int d\theta e^{\sigma\cos(\theta-\theta')}\cos\theta\right]P(\vec{x},\theta',t)$$
$$= \frac{1}{\tau}\int d\vec{x}\int d\theta'\left[\frac{2\pi I_1(\sigma)}{2\pi I_0(\sigma)}\cos(\theta')\right]P(\vec{x},\theta',t)$$
$$= \frac{1}{\tau}\frac{I_1(\sigma)}{I_0(\sigma)}\langle\cos\theta\rangle.$$

This leaves only the run term left:

$$\frac{\partial}{\partial t}\langle\cos\theta\rangle = -\int d\theta(\vec{v}\cdot\vec{\nabla})P\cos\theta - D_r\langle\cos\theta\rangle - \frac{1}{\tau}\left(1-\frac{I_1(\sigma)}{I_0(\sigma)}\right)\langle\cos\theta\rangle.$$

To evaluate this, we can use the fact that we consider an infinite domain, so the probability distribution P must tend to zero at very large x or y:

$$\lim_{x,y\to\pm\infty} P(x,y,\theta,t) = 0.$$

$$\begin{aligned}
&\int d\vec{x}\int d\theta(\vec{v}\cdot\vec{\nabla})P\cos\theta \\
&\qquad = v\int d\theta\int d\vec{x}\left[\cos\theta\frac{\partial}{\partial x}P\cos\theta+\sin\theta\frac{\partial}{\partial y}P\cos\theta\right] \\
&\qquad = v\int d\theta\left[\cos^2\theta\int dy\int dx\frac{\partial P}{\partial x}+\cos\theta\sin\theta\int dx\int dy\frac{\partial P}{\partial y}\right] \\
&\qquad = v\int d\theta\left[\cos^2\theta\int dyP\Big|_{x=-\infty}^{x=\infty}+\cos\theta\sin\theta\int dxP\Big|_{y=-\infty}^{y=\infty}\right] \\
&\qquad = 0
\end{aligned}$$

Finally, we have a simple differential equation for $\langle\cos\theta\rangle$:

$$\frac{\partial}{\partial t}\langle\cos\theta\rangle=-\left(D_r+\frac{1}{\tau}\left(1-\frac{I_1(\sigma)}{I_0(\sigma)}\right)\right)\langle\cos\theta\rangle.$$

This has the exponential solution we were looking for in Equation 4, and we can extract the persistence time as:

$$\tau_p=\frac{1}{D_r+\frac{1}{\tau}\left(1-\frac{I_1(\sigma)}{I_0(\sigma)}\right)}. \tag{5}$$

Short time properties for the run-and-tumble process

In this section, we discuss the short time-scale behavior (ie. the behavior during runs) of the run-and-tumble process, which we can understand by drawing a connection to stochastic resetting processes. The tumbling term in Eq 1 has a sink and source component, analogous to a resetting term [3] (though the resetting affects the orientation instead of the position, as is more typical). With this view, for short timescales (less than the typical length of a run) we can treat this process as a purely active Brownian particle [4, 5]:

$$\frac{\partial}{\partial t}P=-(\vec{v}\cdot\vec{\nabla})P+D_r\frac{\partial^2P}{\partial\theta^2}.$$

We can compute the persistence time of this process as in the previous section:

$$\frac{\partial}{\partial t}\langle\cos\theta\rangle=-D_r\langle\cos\theta\rangle\qquad\rightarrow\qquad\tau_p=D_r^{-1}. \tag{6}$$

By numerically computing the persistence for time-scales less than the mean run time, we can get a value for this rotational diffusion coefficient for the full run-and-tumble process.

We next examine the mean-squared displacement of the active Brownian process [6–8]:

$$\langle r(\Delta t)^2\rangle=\langle\left(\int_0^{\Delta t}\dot{r}(t')dt'\right)^2\rangle.$$

Plugging in the Langevin expression for an active Brownian process, we get:

$$\langle r(\Delta t)^2\rangle=v^2\int_0^{\Delta t}\int_0^{\Delta t}dt_1dt_2\langle\hat{e}(t_1)\cdot\hat{e}(t_2)\rangle.$$

This dot product is essentially the mean orientation used to find the persistence time:

$$\langle r(\Delta t)^2\rangle=v^2\int_0^{\Delta t}\int_0^{\Delta t}dt_1dt_2e^{-D_r(t_2-t_1)}.$$

By making the substitution $\tau = t_2 - t_1$ and noting that the mean orientation is symmetric in τ, one integral becomes trivial and the other can be integrated by parts:

$$\begin{aligned} \langle r(\Delta t)^2 \rangle &= 2v^2 \int_0^{\Delta t} d\tau \int_\tau^{\Delta t} dt_1 e^{-D_r \tau} \\ &= 2v^2 \int_0^{\Delta t} d\tau (\Delta t - \tau) e^{-D_r \tau} \\ &= \frac{2v^2}{D_r^2} \left(D_r \Delta t - 1 + e^{-D_r \Delta t} \right). \end{aligned} \tag{7}$$

Expanding the exponential for short time-scales, we get:

$$\begin{aligned} \langle r(\Delta t)^2 \rangle &= \frac{2v^2}{D_r^2} \left(\frac{D_r^2 \Delta t^2}{2} + \mathcal{O}(\Delta t^3) \right) \\ &\approx v^2 \Delta t^2. \end{aligned} \tag{8}$$

This gives the ballistic behavior we should expect, though it also gives us a way to compute the typical velocity of the process by fitting the short time scale MSD.

References

1. Celani A, Vergassola M. Bacterial Strategies for Chemotaxis Response. Proceedings of the National Academy of Sciences. 2010 Jan;107(4):1391-6. doi:10.1073/pnas.0909673107.

2. Kirkegaard JB, Goldstein RE. The Role of Tumbling Frequency and Persistence in Optimal Run-and-Tumble Chemotaxis. IMA Journal of Applied Mathematics. 2018 Jul;83(4):700-19. doi:10.1093/imamat/hxy013.

3. Gupta S, Jayannavar AM. Stochastic Resetting: A (Very) Brief Review. Frontiers in Physics. 2022 Apr;10. doi:10.3389/fphy.2022.789097.

4. Fodor É, Cristina Marchetti M. The Statistical Physics of Active Matter: From Self-Catalytic Colloids to Living Cells. Physica A: Statistical Mechanics and its Applications. 2018 Aug;504:106-20. doi:10.1016/j.physa.2017.12.137.

5. Moen EQZ, Olsen KS, Rønning J, Angheluta L. Trapping of Active Brownian and Run-and-Tumble Particles: A First-Passage Time Approach. Physical Review Research. 2022 Oct;4(4):043012. doi:10.1103/PhysRevResearch.4.043012.

6. Howse JR, Jones RAL, Ryan AJ, Gough T, Vafabakhsh R, Golestanian R. Self-Motile Colloidal Particles: From Directed Propulsion to Random Walk. Physical Review Letters. 2007 Jul;99(4):048102. doi:10.1103/PhysRevLett.99.048102.

7. ten Hagen B, van Teeffelen S, Löwen H. Brownian Motion of a Self-Propelled Particle. Journal of Physics: Condensed Matter. 2011 Apr;23(19):194119. doi:10.1088/0953-8984/23/19/194119.

8. Löwen H. Inertial Effects of Self-Propelled Particles: From Active Brownian to Active Langevin Motion. The Journal of Chemical Physics. 2020 Jan;152(4):040901. doi:10.1063/1.5134455.
