# Supplementary material for: Stochastic modeling of long-legged ant A. gracilipes locomotion in laboratory experiments
Source: PLoS Comput Biol. 2026 Aug 6;22(8):e1014069. doi: 10.1371/journal.pcbi.1014069 (PMC13446673; doi:10.1371/journal.pcbi.1014069)
Supplement: Supplementary file 2 [file pcbi.1014069.s005.pdf]

Table S1. Parameters for SLEAP ant tracking models.

Parameter	Centroid model	Centered instance model
Architecture	U-net	U-net
Max Stride	16	16
Filters	16	24
Filters Rate	2.0	1.5
Input Scaling	1.0	1.0
Crop Size	N/A	64
Training Augmentation		
Rotation	[-180, 180]	[-180, 180]
Scale	[0.95, 1.05]	[0.95, 1.05]
Contrast	[0.80, 1.40]	[0.80, 1.40]
Brightness	[0.0, 10.0]	[0.0, 10.0]
