# Supplementary material for: Stochastic modeling of long-legged ant A. gracilipes locomotion in laboratory experiments
Source: PLoS Comput Biol. 2026 Aug 6;22(8):e1014069. doi: 10.1371/journal.pcbi.1014069 (PMC13446673; doi:10.1371/journal.pcbi.1014069)
Supplement: Supplementary file 3 [file pcbi.1014069.s006.pdf]

Table S2. Ant tracking trials that are excluded from analysis.

Reason for trial exclusion	Number of trials affected
Specimen has missing or injured leg	2
Specimen has a white pattern on gaster tip, indicating different caste of ant	2
Specimen sits still or barely moves for entire trial	2
Specimen already used for a previous trial	4
Unsatisfactory cleaning procedure left residue throughout arena	5
**Total excluded**	15
**Total analyzed**	95
